# BCR-ABL1-independent PI3Kinase activation causing imatinib-resistance

**DOI:** 10.1186/1756-8722-4-6

**Published:** 2011-02-07

**Authors:** Hilmar Quentmeier, Sonja Eberth, Julia Romani, Margarete Zaborski, Hans G Drexler

**Affiliations:** 1 Leibniz-Institute DSMZ - German Collection of Microorganisms and Cell Cultures, Braunschweig, Germany

## Abstract

**Background:**

The *BCR-ABL1 *translocation occurs in chronic myeloid leukemia (CML) and in 25% of cases with acute lymphoblastic leukemia (ALL). The advent of tyrosine kinase inhibitors (TKI) has fundamentally changed the treatment of CML. However, TKI are not equally effective for treating ALL. Furthermore, *de novo *or *secondary *TKI-resistance is a significant problem in CML. We screened a panel of *BCR-ABL1 *positive ALL and CML cell lines to find models for imatinib-resistance.

**Results:**

Five of 19 *BCR-ABL1 *positive cell lines were resistant to imatinib-induced apoptosis (KCL-22, MHH-TALL1, NALM-1, SD-1, SUP-B15). None of the resistant cell lines carried mutations in the kinase domain of *BCR-ABL1 *and all showed resistance to second generation TKI, nilotinib or dasatinib. STAT5, ERK1/2 and the ribosomal S6 protein (RPS6) are *BCR-ABL1 *downstream effectors, and all three proteins are dephosphorylated by imatinib in sensitive cell lines. TKI-resistant phosphorylation of RPS6, but responsiveness as regards JAK/STAT5 and ERK1/2 signalling were characteristic for resistant cell lines. PI3K pathway inhibitors effected dephosphorylation of RPS6 in imatinib-resistant cell lines suggesting that an oncogene other than *BCR-ABL1 *might be responsible for activation of the PI3K/AKT1/mTOR pathway, which would explain the TKI resistance of these cells. We show that the TKI-resistant cell line KCL-22 carries a PI3Kα E545G mutation, a site critical for the constitutive activation of the PI3K/AKT1 pathway. Apoptosis in TKI-resistant cells could be induced by inhibition of AKT1, but not of mTOR.

**Conclusion:**

We introduce five Philadelphia-chromosome positive cell lines as TKI-resistance models. None of these cell lines carries mutations in the kinase domain of *BCR-ABL1 *or other molecular aberrations previously indicted in the context of imatinib-resistance. These cell lines are unique as they dephosphorylate ERK1/2 and STAT5 after treatment with imatinib, while PI3K/AKT1/mTOR activity remains unaffected. Inhibition of AKT1 leads to apoptosis in the imatinib-resistant cell lines. In conclusion, Ph+ cell lines show a form of imatinib-resistance attributable to constitutive activation of the PI3K/AKT1 pathway. Mutations in *PIK3CA*, as observed in cell line KCL-22, or PI3K activating oncogenes may undelie TKI-resistance in these cell lines.

## Background

Expression of the Philadelphia chromosome (Ph), resulting from fusion of the non-receptor tyrosine kinase *ABL1 *on chromosome 9 with *BCR *on chromosome 21, is the hallmark of chronic myeloid leukemia (CML), but is also found in 20-30% of acute lymphoblastic leukemia (ALL) cases. The development of clinically applicable tyrosine kinase inhibitors (TKI) has fundamentally changed the treatment of patients with CML: imatinib mesylate induces hematologic remission in nearly all CML patients [1]. In Ph+ ALL, imatinib is much less effective [2]. Causes for imatinib-resistance are (i) the development of cell clones carrying mutations in the kinase domain of *BCR-ABL1 *[3,4]; (ii) low intracellular drug levels caused by disordered expression of influx and efflux transporters [5,6]; (iii) overexpression of *BCR-ABL1 *[7,8]; and (iv) activation of alternative signalling pathways by oncogenic enzymes like v-src sarcoma viral oncogene homolog (SRC) kinases [9,10] or guanosine triphosphatases (GTPases) [11].

Many studies performed to elucidate imatinib-resistance have made use of cells ectopically expressing *BCR-ABL1 *or of cell lines which gained resistance after prolonged exposure to rising drug concentrations [6,7]. Cell lines that were inherently imatinib-resistant have rarely been used, which is astonishing because imatinib-resistant cell lines KCL-22 and SD-1 were described very early, in 1997 [12]. Here, we screened the DSMZ cell lines bank to find imatinib-resistant *BCR-ABL1 *positive cell lines. Five out of 19 Ph+ cell lines (26%) were resistant to imatinib. We set out to investigate whether these cell lines displayed the known molecular and cellular causes for imatinib-resistance.

## Results and Discussion

### Imatinib-resistant *BCR-ABL1 *positive cell lines

**Figure 1:**
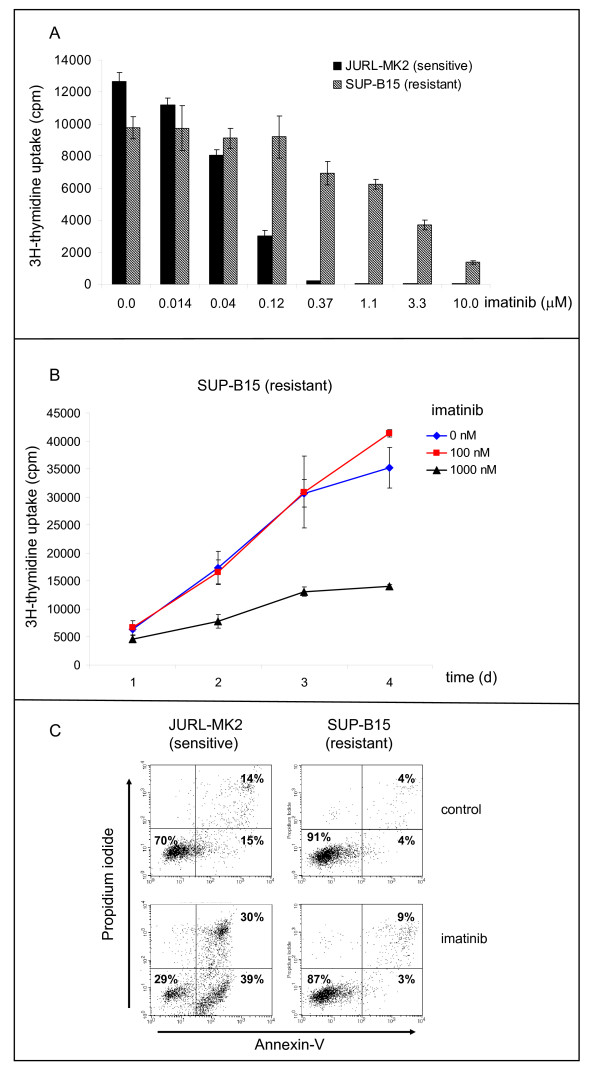
**Imatinib-responsive and -resistant *BCR-ABL1 *positive cell lines**. A) [^3^H]-thymidine incorporation after 24 h incubation with imatinib. Results of cell line JURL-MK2 are representative for imatinib-sensitive, results of cell line SUP-B15 are representative for imatinib-resistant cell lines. B) Time-course data confirm resistance of cell line SUP-B15 to imatinib (100 nM) for a period of four days. C) Apoptosis assessed by annexin-V/PI staining. Imatinib (1 μM, 24 h) induced apoptosis in sensitive JURL-MK2 cells, but not in the imatinib-resistant cell line SUP-B15. Control: cells cultivated for 24 h in medium without TKI.

A panel of Ph+ ALL and CML cell lines was tested in [^3^H]-thymidine and annexin-V/propidium iodide (PI) assays to find models for TKI-resistance studies (Figure 1). In 14/19 *BCR-ABL1 *positive cell lines, IC50 values for imatinib were in the range of 50 nM to 200 nM. Five cell lines showed markedly higher IC50 values: KCL-22 (800 nM), MHH-TALL1 (1 μM), NALM-1 (> 10 μM), SD-1 (> 10 μM), and SUP-B15 (2 μM) (Table 1). These cell lines were inherently resistant to imatinib according to the results of proliferation and apoptosis assays, as they had not been preincubated with the TKI.

**Table 1:** TKI-resistance of *BCR-ABL1*-positive cell lines

**CML**	**stage**	**IC50 imatinib (nM)**	**IC50 nilotinib (nM)**	**IC50 dasatinib (nM)**	***BCR-ABL1 *mu/wt**	***BCR-ABL1 *breakpoint**	***Ikaros *Ik6**	***BCR-ABL1 *mRNA** **expression level**
		
BV-173	B BC	100	< 10	n.d.	n.d.	b2-a2	yes	1.6
CML-T1	T BC	200	20	n.d.	n.d.	b2-a2	no	0.5
EM-2	M BC	80	< 10	n.d.	wt	b3-a2	no	n.d.
HNT-34	M BC	100	10	n.d.	n.d.	b3-a2	no	n.d.
JK-1	M BC	100	10	n.d.	n.d.	b2-a2	no	1
JURL-MK1	M BC	200	< 10	< 1	n.d.	b3-a2	no	n.d.
JURL-MK2	M BC	50	< 10	< 1	n.d.	b3-a2	no	n.d.
K-562	M BC	200	20	n.d.	n.d.	b3-a2	no	n.d.
**KCL-22**	M BC	800	40	1	wt	b2-a2	no	1.7
KU-812	M BC	50	< 10	n.d.	n.d.	b3-a2	no	n.d.
KYO-1	M BC	80	< 10	< 1	n.d.	b2-a2	no	0.9
LAMA-84	M BC	100	< 10	< 1	n.d.	b3-a2	no	n.d.
MEG-01	M BC	200	< 10	< 1	n.d.	b2-a2	no	n.d.
MOLM-6	M BC	50	< 10	< 1	n.d.	b2-a2	no	n.d.
**NALM-1**	B BC	> 10000	5000	> 1000	wt	b2-a2	no	1
								
**pre B-ALL**								
**SD-1**	B lymph	> 10000	2000	n.d.	wt	e1-a2	no	0.1
**SUP-B15**	pre B	2000	500	100	wt	e1-a2	yes	1.1
TOM-1	pre B	80	5	n.d.	n.d.	e1-a2	no	1
								
**T-ALL**								
**MHH-TALL-1**	T-ALL	1000	1000	n.d.	wt	e6-a2	no	n.d.

IC50 values for TKI imatinib, nilotinib and dasatinib were determined by [^3^H]-thymidine uptake 24 h after onset of incubation with varying concentrations of the individual inhibitors (bold: TKI-resistant cell lines). Note that imatinib-resistant cell lines were also resistant to second-generation inhibitors nilotinib and dasatinib. The *BCR-ABL1 *kinase domain of TKI-resistant cell lines was sequenced and found to be wild-type (wt). Expression of *Ikaros *splice variant 6 (Ik6) was determined by conventional PCR. *BCR-ABL1 *mRNA expression levels in cell lines with different breakpoints (b2-a2; e1-a2) were determined with quantitative real-time PCR. *BCR-ABL1 *expression of cell line JK-1 was set to 1 for b2-a2 positive cell lines; for e1-a2 positive cell lines, TOM-1 was the reference cell line. B-cell BC: B blast crisis; B lymph: B lymphoblastoid; T BC: T-cell blast crisis; M BC: myeloid blast crisis; mu: mutant; wt: wild-type; n.d.: not done.

### *BCR-ABL1 *mutations, *BCR-ABL1 *expression, imatinib transporters

Point mutations in the kinase domain of *BCR-ABL1 *are the main cause of imatinib-resistance in the chronic phase of CML [13]. Although second generation *BCR-ABL1 *inhibitors (nilotinib, dasatinib) are effective in most *BCR-ABL1 *mutated cases, all 5 imatinib-insensitive cell lines identified here were also resistant to nilotinib suggesting that resistance might not be caused by *BCR-ABL1 *mutations (Table 1). In accordance with this notion, genomic sequencing showed no sequence alterations in the kinase domain of the resistant cell lines (Table 1).

The DNA-binding protein Ikaros is a major regulator of lymphoid development [14]. Deletion of *Ikaros *is found in the majority of *BCR-ABL1*-positive ALL and of CML in progression to lymphoid blast crisis [15,16]. Public genomic array data indicate hemizygous loss of the 7p12 region in cell line NALM-1, including *IKZF1 *and the neighbouring gene *Dopa decarboxylase *(*DDC*) http://www.sanger.ac.uk/cgi-bin/genetics/CGP/10kCGHviewer.cgi?chr=7&dna=NALM-1. Genomic PCR analysis confirmed loss of *IKZF1 *in this cell line, but not in cell lines SD-1, SUP-B15 and MHH-TALL-1 (Additional File 1). However, the majority of Ph+ ALL with *IKZF1 *aberrations do not show deletion of the whole gene, but instead intragenic loss of various *IKZF1 *exons, leading to the expression of mRNA variants that mimic "normal" splice variants [15,16]. A recent publication correlates expression of the *Ikaros *variant Ik6 with high *BCR-ABL1 *mRNA levels and imatinib-resistance in Ph+ ALL [17]. We could not confirm this correlation among Ph+ ALL and CML cell lines: Ik6 was expressed in 2/19 *BCR-ABL1 *positive cell lines, one being imatinib sensitive (BV-173) and one resistant (SUP-B15) (Table 1). Neither cell line SUP-B15 nor most other TKI-resistant cell lines showed particularly high *BCR-ABL1 *expression levels according to quantitative RT-PCR analysis (Table 1). The only exception was cell line KCL-22 with about 2-fold higher *BCR-ABL1 *expression levels, both at the mRNA (Table 1) and the protein level (not shown). While supporting the notion that a causative correlation might exist between the high expression of the mutated kinase and imatinib-resistance for cell line KCL-22, these results also showed that in 4/5 cell lines TKI-resistance was not the consequence of *BCR-ABL1 *overexpression (Table 1).

**Figure 2:**
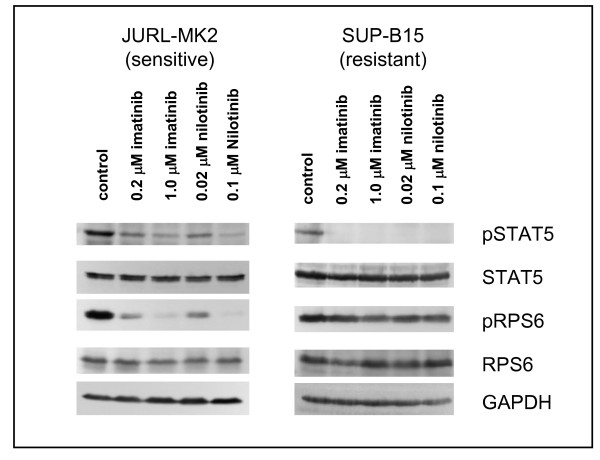
**Effect of tyrosine-kinase inhibitors on phosphorylation of STAT5 and RPS6 in TKI-sensitive and -resistant cell lines**. Cell lines JURL-MK2 and SUP-B15 were treated for 3 h with imatinib or nilotinib. Phosphorylation of STAT5 and RPS6 was determined by Western blot analysis using the appropriate antibodies. Note that imatinib and nilotinib inhibited STAT5 phosphorylation (as shown by loss of pSTAT5) in TKI-sensitive and -resistant cell lines. GAPDH was used as loading control.

Thus, neither *BCR-ABL1 *mutations nor overexpression of the kinase were the general cause for imatinib-resistance in these cell lines. Further analyses showed that also dysregulation of drug transporters was improbable: unlike imatinib, nilotinib is neither imported via hOCT-1, nor exported via ABCB1 [5,18]. All five imatinib-resistant cell lines were nilotinib-resistant (Table 1). Therefore, it appeared unlikely that imatinib-resistance was caused by deregulated transport proteins. Finally, the finding that both imatinib and nilotinib induced dephosphorylation of signal transducer and activator of transcription 5 (STAT5) in the TKI-resistant cell line SUP-B15 as shown in Figure 2 further excludes resistance being due to low intracellular drug levels. Both drugs were transported into the cells which responded by dephosphorylating STAT5 while retaining viability.

### SRC kinases

**Figure 3:**
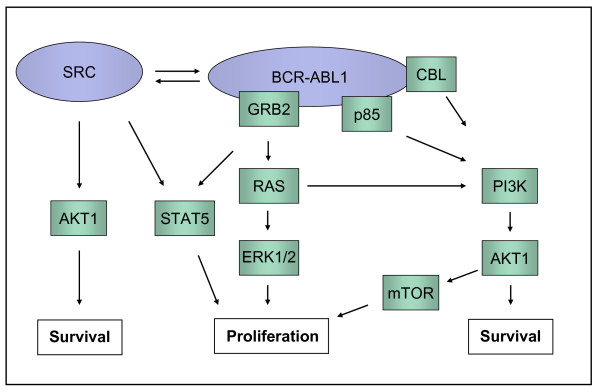
**BCR-ABL1 signalling cascades**. BCR-ABL1 induces activation of JAK2/STAT5, RAS/RAF/ERK and PI3K/AKT1/mTOR signalling pathways. SRC kinase family members may interact with BCR-ABL1 leading to mutual activation. The scheme has been adopted [11,18,19]. RPS6 is a downstream target of AKT1/mTOR/p70S6K.

SRC kinases had been described to play an important role in *BCR-ABL1 *positive ALL (Figure 3) [9,19-21]. Interestingly, 4/5 imatinib-resistant Ph+ cell lines were from patients with pre-B ALL, T-ALL, or CML in B-cell blast crisis (Table 1). Among lymphoid Ph+ cell lines 5/7 were imatinib-resistant, including TOM-1, a pre-B cell line classed "semiresistant" displaying normal IC50 values in the thymidine uptake assay while remaining relatively unresponsive to higher concentrations (Table 1). Therefore, we applied dasatinib to elucidate whether activity of SRC kinases was important for the growth of imatinib-resistant cells. Dasatinib is a dual BCR-ABL1 and SRC kinase inhibitor, as evidenced by its ability to inhibit phosphorylation of SRC and STAT5 in TKI-responsive JURL-MK2 cells (Additional File 2). However, two of three imatinib-resistant cell lines tested (NALM-1, SUP-B15) were resistant to dasatinib in the proliferation assay (Table 1). Furthermore, TKI-resistant SUP-B15 cells did not express an active, phosphorylated SRC kinase and dasatinib did not affect RSP6 phosphorylation in this cell line (Additional File 2). These results are not consistent with the notion that SRC kinases are the cause of imatinib-resistance in these cell lines.

### Imatinib induces dephosphorylation of ERK1/2 and of STAT5 in TKI-resistant cell lines

*BCR-ABL1 *positive cells are characterized by stimulation of the Janus kinase 2 (JAK2)/STAT5, extracellular-signal-regulated-kinase (ERK) 1/2 and phosphoinositide-3-kinase/v-Akt murine thymoma viral oncogene homolog 1/mammalian target of rapamycin (PI3K/AKT1/mTOR) pathways (Figure 3) [21-23]. To determine the activity of these signalling cascades, we assessed the phosphorylation status of STAT5, ERK1/2 and of the mTOR complex 1 (mTORC1) substrate ribosomal S6 protein (RPS6).

**Figure 4:**
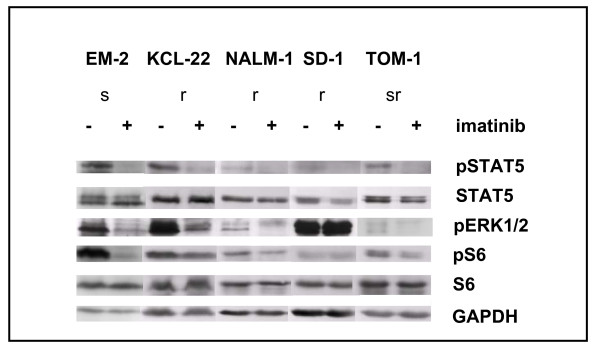
**Phosphorylation levels of STAT5, ERK1/2 and RPS6 in TKI-sensitive and -resistant cell lines**. Cell lines were treated for 3 h with/without imatinib (200 nM). Phosphorylation of STAT5, ERK1/2 and RPS6 was determined by Western blot analysis. Note that imatinib spares or only marginally induces dephosphorylation of RPS6 in imatinib-resistant cell lines. ERK1/2 dephosphorylation is seen in most imatinib-resistant cell lines. Abbreviations: s TKI-sensitive; r TKI-resistant; sr semi-resistant.

In TKI-sensitive cells, imatinib induced dephosphorylation of all three proteins (Figure 4). In TKI-resistant cell lines, treatment with TKI reduced phosphorylation of STAT5 (5/5 cell lines) and of ERK1/2 (4/5 cell lines) but did not comparably affect phosphorylation of RPS6 (Figure 2 Figure 4). This observation allowed three conclusions: (i) cells that survive in the presence of imatinib are not necessarily completely unresponsive to the drug; (ii) activation of ERK1/2 and the JAK/STAT5 pathway is not obligatory for short-term proliferation of Ph+ positive cell lines; (iii) TKI-resistance is correlated with - if not actually caused by - the constitutive and imatinib-resistant activity of the PI3K/AKT1/mTOR pathway.

### *BCR-ABL1*-resistant cell lines show constitutive activation of mTORC1

The PI3K/AKT1/mTOR/p70S6kinase (p70S6K) pathway is a *BCR-ABL1 *downstream target and implicated in the survival of leukemic cells (Figure 3) [23,24]. A major difference between TKI-sensitive and -resistant cell lines was seen with respect to the phosphorylation level of the p70S6K substrate RPS6: incubation with imatinib inhibited RPS6 phosphorylation in TKI-responsive, but not - or to a much lesser degree - in TKI-resistant cell lines (Figure 2 Figure 4). p70S6K is an exclusive substrate of mTOR complex 1 (mTORC1). Rapamycin inhibits this complex, but not mTORC2 [25]. Recent studies suggest that targeting mTOR might become an efficient anti-cancer therapy [25]. Rapamycin arrests Ph+ K-562 cells in the G1 phase of the cell cycle and induces apoptosis in primary CML cells [26]. Antileukemic effects of rapamycin in patients with TKI-resistant CML have been shown [27]. These results prompted us to test whether rapamycin inhibits constitutive RPS6 phosphorylation, whether it reduces cell growth of TKI-resistant CML cell lines and - most importantly - whether the combination of rapamycin and imatinib induces apoptosis in imatinib-resistant cells.

**Figure 5:**
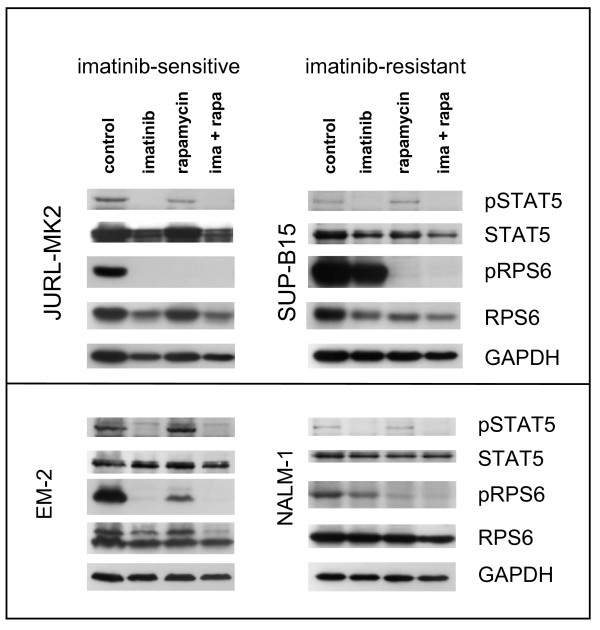
**Effect of imatinib and rapamycin on phosphorylation of STAT5 and RPS6**. Cell lines were treated for 24 h with imatinib (1 μM), rapamycin (10 nM) or a combination of both agents. Phosphorylation of STAT5 and RPS6 was determined by Western blot analysis. Note that imatinib induces dephosphorylation of STAT5 and of RPS6 in TKI-sensitive cell lines, dephosphorylation of STAT5 in TKI-resistant cell lines. Rapamycin inhibits phosphorylation of RPS6; ima = imatinib; rapa = rapamycin.

**Figure 6:**
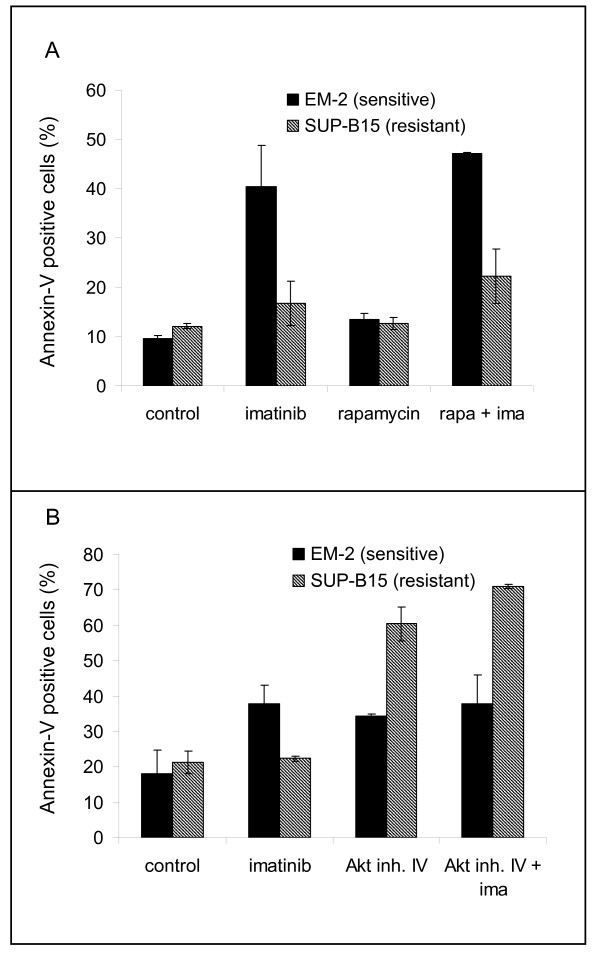
**Inhibition of AKT1, but not of mTORC1 induces apoptosis in imatinib-resistant cell lines**. Apoptosis was assessed by Annexin-V staining. Experiments were performed in triplicates. Cell lines were treated for 24 h with A) imatinib (1 μM), rapamycin (10 nM), or a combination of both agents. Note that rapamycin did not induce apoptosis and did not sensitize resistant cells to imatinib. Additional experiments showed that preincubation with rapamycin did not sensitize resistant cells, as well. B) Imatinib (1 μM), Akt inhibitor IV (1 μM), or both agents were applied for 24 h; additional experiments showed that 1 μM Akt inhibitor IV induced apoptosis also in cell lines BV-173 (imatinib-sensitive) and NALM-1 (imatinib-resistant).

Rapamycin effected dephosphorylation of RPS6 in imatinib-sensitive and imatinib-resistant cell lines (Figure 5). Rapamycin alone did not induce apoptosis in imatinib-resistant cell lines, as evidenced by annexin-V staining (Figure 6A). However, in 6/6 cell lines, rapamycin (10 nM, for 24 h) reduced [^3^H]-thymidine uptake, which was paralleled by an increase in the percentage of G1-phase cells (Table 2). For multiple myeloma, it has been shown that an antiproliferative drug, the CDK4/6 inhibitor PD0332991 can sensitize cells to a second agent, a cytotoxic drug (bortezomib) [28]. Therefore, we speculated that rapamycin and imatinib might cooperate in a similar way, rapamycin acting as growth inhibitor and imatinib as cytotoxic agent. The combination of rapamycin plus imatinib had the same inhibitory effect on phosphorylation of RPS6 and of STAT5 in TKI-resistant cells as imatinib alone had in TKI-sensitive cells (Figure 5). However, the combination of imatinib and rapamycin did not lead to a significant increase of apoptotic cells in imatinib-resistant cells, compared to the effects of each drug alone (Figure 6A). Thus, inhibition of mTORC1 was insufficient to restore responsiveness in TKI-resistant cell lines.

**Table 2:** Effect of rapamycin on proliferation of *BCR-ABL1*-positive cell lines

	**[^3^H]-thymidine (SI)**		**% cells in G1**	
	**control**	**rapamycin**	**control**	**rapamycin**
	
**TKI-sensitive**				
BV-173	1	0.3 +/- 0.1	63 +/- 4	69 +/- 4
EM-2	1	0.5 +/- 0.1	61 +/- 2	68 +/- 1
JURL-MK2	1	0.6 +/- 0.1	73 +/- 1	81 +/- 2
				
**TKI-resistant**				
KCL-22	1	0.4 +/- 0	60 +/- 5	74 +/- 2
NALM-1	1	0.6 +/- 0.1	76 +/- 2	80 +/- 1
SUP-B15	1	0.5 +/- 0.1	66 +/- 1	83 +/- 2

Proliferation was assessed applying the [^3^H]-thymidine incorporation assay. Stimulation index (SI) was determined setting uptake (cpm) of untreated cells to 1. Shown are results of three experiments, each done in triplicate. Cell cycle analysis was performed by flow cytometry with ethanol-fixed, PI stained cells. Experiments were performed in triplicates. Both assays were performed after 24 h with/without rapamycin (10 nM).

### AKT1, mediator of imatinib-induced apoptosis

As shown in this study, 2/3 BCR-ABL1 downstream signalling cascades - the JAK2/STAT5 and the ERK1/2 pathways - are druggable by TKI in imatinib-resistant cell lines (Figure 4). The PI3K/mTOR pathway was not comparably inactivated by imatinib, as assessed by RPS6 phosphorylation (Figure 2 Figure 4). These results imply that TKI-resistance is caused by constitutive TKI-unresponsive activation of the PI3K/mTOR pathway. However, rapamycin - despite efficiently dephosphorylating RPS6 - failed to induce apoptosis, whether alone or in combination with imatinib (Figure 6A). Therefore, we concluded that another member of the PI3K pathway, upstream of mTOR might confer resistance, inhibiting imatinib-triggered apoptosis. It has been shown in another experimental setting that the inhibition of the serine-threonine kinase AKT1 sensitizes tumor cells to apoptotic stimuli [29]. AKT1 stimulates proliferation by activation of mTORC1, and suppresses apoptosis by phosphorylation of proapoptotic proteins like BCL2-associated agonist of cell death (BAD) (Figure 3). We inhibited AKT1 with Akt inhibitor IV, as evidenced by dephosphorylation of RPS6 (Additional File 3). Inhibition of AKT1 triggered apoptosis in imatinib-sensitive and -resistant cell lines (Figure 6B). These data suggest that AKT1, rather than mTOR is the PI3K pathway member that should be inhibited to trigger apoptosis in TKI-resistant cells.

### Role of PI3Kα in imatinib-resistance in Ph+ cell lines remains elusive

In this study we show that imatinib-resistance of Ph+ cell lines may be ascribed to the TKI-insensitive activation of the PI3K/AKT1/mTOR pathway. Although other BCR-ABL1-triggered signalling cascades (ERK1/2, JAK2/STAT5) proved to be imatinib-responsive, inhibition of these pathways did not affect the viability of cells. In contrast to imatinib, wortmannin (PI3K inhibitor), OSU-03102 (PDK1 inhibitor) and rapamycin (mTOR inhibitor) inhibited the PI3K/AKT1/mTOR pathway, suggesting that the TKI-resistance observed in the Ph+ cell lines might be caused by a PI3K-activating oncogene other than BCR-ABL1 itself (Additional File 4). To identify this oncogene we looked for mutations and aberrant expression of genes known to mediate activation of PI3K, such as *RAS*, *CBL *and *p85 *(Figure 3). In addition, *PI3K *itself was a candidate for genetic alterations causing constitutive activation of the PI3K/AKT1 pathway.

*RAS *mutations occur quite frequently in hematologic malignancies (5% *K-RAS*, 12% *N-RAS*) [30]. However, none of the TKI-resistant cell lines showed mutations of the most affected regions of the genes (amino acids 12, 13 and 60 in *K-RAS*, amino acids 12, 13, and 61 in *N-RAS*; data not shown), a finding which was scarcely unexpected because *RAS *mutations would not only stimulate PI3K, but also ERK1/2 in an imatinib-insensitive manner (Figure 3). However, ERK1/2 was silenced by imatinib in 4/5 cell lines (Figure 2 Figure 4).

The *PI3K *subunit *p85β *(*PIK3R2*) and the *Casitas B-Cell lymphoma gene *(*CBL*) belong to those seven genes identified as core components for coordinating the oncogenic functions of BCR-ABL1 [21]. Phosphorylation of CBL recruits the p85 subunit of PI3K leading to activation of PI3K/AKT1/mTOR pathway [31]. Quantitative RT-PCR did not reveal major differences in the expression of *CBL *and *p85 *between imatinib-sensitive and -resistant cell lines (data not shown). Besides, we did not detect alterations in exons 7-9 of *CBL*, described as transforming mutations in myeloid malignancies (data not shown) [31,32].

**Figure 7:**
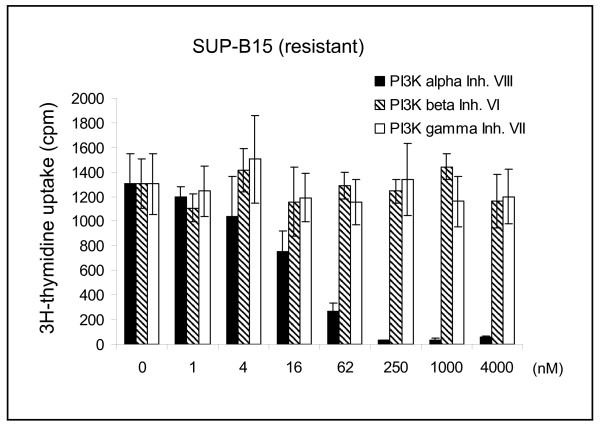
**PI3Kα is important for cell growth of Ph+ cell lines**. [^3^H]-thymidine incorporation after 24 h incubation with inhibitors for PI3Kα, PI3Kβ and PI3Kγ in cell line SUP-B15. Experiments with cell lines NALM-1 (resistant) and BV-173 (sensitive) yielded similar results, confirming the importance of PI3Kα for growth of Ph+ cell lines.

**Figure 8:**
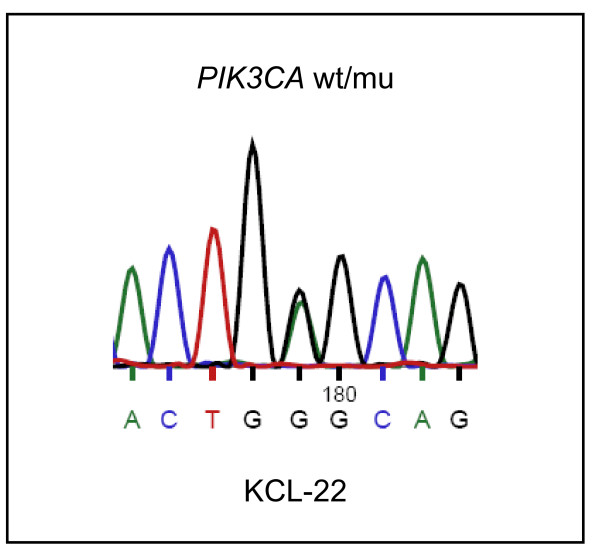
**PI3Kα E545G mutation in cell line KCL-22**. Genomic sequencing and cDNA sequencing (shown here) showed that cell line KCL-22 heterozygously carried PI3Kα E545G. No mutations were found in the helical (exon 10) and kinase domains (exon 21) of the remaining TKI-resistant cell lines.

Class I PI3Ks are heterodimeric proteins consisting of a catalytic and a regulatory adaptor subunit (e.g. p85β). To find out which specific PI3K might be involved in imatinib-resistant activation of AKT1/mTOR, we applied inhibitors with differing specificities for the various PI3K catalytic subunits. [^3^H]-thymidine incorporation data suggested that PI3Kα (PIK3CA), but not PI3K β or PI3Kγ play a role in the imatinib-resistance of the cell lines tested (Figure 7). Mutations occurring in the catalytic subunit *PIK3CA *result in constitutive activation and oncogenicity [33]. The majority of *PIK3CA *mutations occur either in the helical (exon 10) or in the kinase domain (exon 21) of the gene [34]. Thus, we sequenced the respective regions of *PIK3CA *(database number ENST000000263967) in all imatinib-resistant cell lines. We did not find mutations in the kinase domain, but cell line KCL-22 carried a heterozygous point mutation in the helical domain, leading to the amino acid change PI3Kα E545G (Figure 8). PI3Kα E545 mutations have been observed in clinical samples of solid tumors and the E545A mutation has been shown to constitutively activate the PI3K pathway [33].

These data suggest that also the PI3Kα E545G mutation that we identified in cell line KCL-22 may be responsible for the constitutive activity of the PI3K/AKT1 pathway conferring TKI-resistance to the cells. Deep sequencing might help to elucidate whether activating mutations in oncogenes other than *BCR-ABL1 *or *PIK3CA*, or loss of tumor suppressor genes trigger the PI3K in cell lines NALM-1, SD-1, SUP-B15 and MHH-TALL1, thus causing TKI-resistance.

## Conclusion

In this study an unexpectedly high number (5/19, 26%) of Ph+ ALL and CML cell lines tested imatinib-resistant. The unresponsiveness of the cell lines was not attributable to known causes as *BCR-ABL1 *mutations or activation of SRC kinases. While the *BCR-ABL1*-triggered JAK2/STAT5 and ERK1/2 pathways were inhibited by imatinib, the resistant cell lines stand out by the constitutive activation of the PI3K/AKT1/mTOR pathway. The mTOR inhibitor rapamycin inhibited cell growth, but did not induce apoptosis and did not sensitize resistant cells to imatinib. Instead, inhibition of AKT1 induced apoptosis in TKI-resistant cell lines. Cell line KCL-22 carries a heterozygous mutation in the helical domain of *PIK3CA*, a site critical for activation of the gene. These results suggest that activating mutations in the PI3K itself or in PI3K-stimulating oncogenes might be the molecular cause for TKI resistance.

## Methods

### Human cell lines

The cell lines applied in this study were taken from the stock of the cell bank (DSMZ - German Collection of Microorganisms and Cell Cultures) or were provided by originators. Detailed references and cultivation protocols have been described previously [35].

### Inhibitors

Imatinib and nilotinib were generously provided by Novartis (Basel, Swizerland). Ten mM stock solutions were prepared in H_2_O (imatinib) or DMSO (nilotinib). Dasatinib (100 mM in DMSO) was obtained from LC Laboratories (Woburn, MA, USA). The SRC inhibitor SU 6656 (40 mM in DMSO) was obtained from Cayman Chemical (Ann Arbor, MI, USA). Rapamycin (100 μM in DMSO) was purchased from Cell Signalling (New England Biolabs, Frankfurt, Germany). Akt inhibitor IV, Akt inhibitor VIII, PI3Kα inhibitor VIII, PI3Kβ inhibitor VI, PI3Kγ inhibitor VII and Raf1 kinase inhibitor I (each 10 mM in DMSO) were purchased from Merck (Nottingham, GB). OSU-03012 (100 mM in DSMO) was obtained from Tebu-bio (Offenbach, Germany). All solutions were stored at -20°C.

### [^3^H]-Thymidine uptake, cell cycle analysis and detection of apoptotic cells

Assays of [^3^H]-thymidine incorporation were executed as follows: 1.25 × 10^4 ^cells (in 100 μl) were seeded in triplicate in 96-well flat-bottom microtiter plates. Inhibitors were added as 2x concentrated solution in a 100 μl volume. For the last 3 h of the incubation period, 1 μCi [^3^H]-thymidine (Hartmann Analytic, Braunschweig, Germany) was added to each well. Apoptotic cells were detected and quantified with the annexin-V/PI method using the TACS Annexin-V-FITC kit (R&D Systems, Wiesbaden, Germany) according to the manufacturer's instructions. Binding of fluorescein isothiocyanate-labeled annexin-V and PI staining of the cells was determined by flow cytometry on the FACSCalibur (Becton Dickinson, Heidelberg, Germany). For cell cycle analysis, cells were fixed with 70% ethanol (-20 °C, 20 min on ice), washed with phosphate-buffered saline, and stained with PI (20 μg/ml). DNA content of the cells was determined by flow cytometry.

### Sequencing of the *BCR-ABL1 *kinase domain, of *CBL *exons 7-9 and of *PIK3CA *exons 10 and 21

Exclusively to amplify the kinase domain of *BCR-ABL1*, hemi-nested PCR was performed according to Hochhaus et al. [36]. For cell lines carrying b2-a2 and b3-a2 *BCR-ABL1 *fusion, the following primers were used in first-round PCR: *BCR *exon 13 forward: 5'-ACA GCA TTC CGC TGA CCA TCA ATA AG-3'; *ABL1 *exon 7 reverse (A7-): 5'-AGA CGT CGG ACT TGA TGG AGA ACT-3'. For cell lines with e1-a2 and e6-a2 *BCR-ABL1 *translocation, the same *ABL1 *exon 7 reverse primer (A7-) was combined with the *BCR *exon 1 forward primer: 5'-CCC CCG GAG TTT TGA GGA TTG C-3' [37]. First-round PCRs were performed at 60 °C, respectively 59 °C for 35 cycles. The PCR products were diluted (1/10^6^) and applied in a second-round PCR at 59 °C for 25 cycles using reverse primer A7- and the *ABL1 *exon 4 forward primer: 5'-TGG TTC ATC ATC ATT CAA CGG TGG-3'. Purified PCR products were sequenced using the second-round primers.

The following primers were used to amplify and to sequence *CBL *exons 7-9 from cDNA. *CBL *exon 6 forward: 5'-TCC CTC ACA ATA AAC CTC TCT TCC-3'; *CBL *exon 10 reverse primer: 5'-GCC ATG GAG AAT GGA GAA GGC-3'. RT-PCR was performed for 32 cycles with an annealing temperature of 56°C. Primers for genomic *PIK3CA *PCR were: *PIK3CA *intron 9/10 forward: 5'-GAT TGG TTC TTT CCT GTC TCT TG-3'; *PIK3CA *intron 10/11 reverse: 5'-CCA CAA ATA TCA ATT TAC AAC CAT TG-3'; *PIK3CA *intron 20/21 forward: 5'-TGA CAT TTG AGC AAA GAC CTG; *PIK3CA *exon 21 reverse: 5'-TGG ACT TAA GGC ATA ACA TG-3'. Primers for *PIK3CA *RT-PCR were: *PIK3CA *exon 9 forward: 5'-TGG AGT TTG ACT GGT TCA GC-3', *PIK3CA *exon 11 reverse: 5'-GGG TAG AAT TTC GGG GAT AG-3'. PCR was performed at 55°C for 35 cycles.

### Quantitative real-time PCR analysis

Quantitative PCR was performed on a 7500 Applied Biosystems (Darmstadt, Germany) real-time PCR system using the manufacturer's protocol. RNA was prepared using the RNeasy Mini kit (Qiagen, Hilden, Germany). For mRNA quantification, reverse transcription was performed using the SuperScript II reverse transcriptase kit (Invitrogen, Karlsruhe, Germany). Expression of *BCR-ABL1 *e1-a2 and b2-a2 fusion mRNAs, of *CBL *and of *p85*β (*PIK3R2*) were assessed using the SYBR GREEN PCR Master Mix (Applied Biosystems) with *GAPDH *as internal control. *BCR *(e1) forward: 5'-GCA AGA CCG GGC AGA TCT G-3'; *BCR *(b2) forward: 5'-CAT TCC GCT GAC CAT CAA TAA G-3'; *ABL1 *(a2) reverse: 5'-AGA TGC TAC TGG CCG CTG A-3'; *CBL *forward: 5'-ACC ATA AGC CTC TTC AAG GAG-3'; *CBL *reverse: 5'-AGA TGA GGG ACA GTT TGG TTA G-3'; *GAPDH *forward: 5'-TGG GTG TGA ACC ATG AGA AG-3'; *GAPDH *reverse: 5'-TCC ACG ATA CCA AAG TTG TCA-3'; *p85 *forward: 5'-CAG TCC TCC CCA CCT GAT GT-3'; *p85 *reverse: 5'-GCG GTA GTG AGA TTC GCT GT-3'. Relative expression levels were calculated using the ΔΔCt-method.

### Expression analysis of *Ikaros *splice variant 6 (Ik6)

For detection of *Ikaros *splice variant 6 (Ik6), we performed PCR using the following primers: *Ikaros *exon 2 forward: 5'-ATG GAT GCT GAT GAG GGT CAA GAC-3'; *Ikaros *exon 8 reverse: 5'-GAT GGC TTG GTC CAT CAC GTG G-3'. The PCR was performed with an annealing temperature of 62°C. Splice variants were detected by electrophoresis on a 1.2% agarose gel and verified by sequencing of the PCR products.

### Western blot analysis

Samples were prepared as described previously [38]. The anti STAT5 monoclonal antibody (mAb) was purchased from BD Transduction Laboratories (Heidelberg, Germany). Anti pSTAT5, anti pRPS6 and anti pSrc (Tyr416) antisera as well as the monoclonal antibody directed against RPS6 were purchased from Cell Signalling (New England Biolabs, Frankfurt, Germany). Anti FYN and anti LYN antisera were obtained from Santa Cruz (Heidelberg, Germany). The anti GAPDH mAb was purchased from Abcam (Cambridge, UK). Specific bands on nitrocellulose membranes were visualized with the biotin/streptavidin-horseradish peroxidase system (Amersham, Freiburg, Germany) in combination with the "Renaissance Western Blot Chemoluminescence Reagent" protocol (Perkin Elmer, Waltham, MA, USA).

## Competing interests

The authors declare that they have no competing interests.

## Authors' contributions

HQ designed the study and wrote the manuscript, SE participated in data analysis, JR performed Western blot analysis and [^3^H]-thymidine uptake analysis, MZ performed quantitative real-time PCR analysis and flow-cytometry, HGD provided cell lines and critically read the manuscript. All authors read and approved the final manuscript.

## Authors' information

Leibniz-Institute DSMZ - German Collection of Microorganisms and Cell Cultures, Braunschweig, Germany

## Supplementary Material

Additional file 1 **Deletion of *IKZF1 *in cell line NALM-1**. Quantitative genomic PCR confirmed loss of the genes *IKZF1 *and *DDC*, located between *ELMO-1 *and *ADAM-22 *at chromosome 7p12.2. The cytogenetically-verified diploid B-lymphoblastoid cell line NC-NC was used as reference, the repetitive element *LINE1 *was used as endogenous control. Cell lines SD-1, SUP-B15 and MHH-TALL1 did not show loss of *IKZF1 *according to quantitative PCR. Click here for file

Additional file 2 **Phosphorylation levels of SRC, STAT5 and RPS6 in TKI-sensitive (JURL-MK2) and -resistant (SUP-B15) cell lines**. Cell lines were treated for 3 h with the BCR-ABL1 and SRC kinase inhibitor dasatinib (20 nM) or with the SRC kinase inhibitor SU-6656 (2 μM) or control. Phosphorylation of the SRC kinases Fyn and Lyn, of STAT5 and RPS6 was determined by Western blot analysis. Click here for file

Additional file 3 **Phosphorylation levels of RPS6 in TKI-sensitive and -resistant cell lines**. Cell lines were treated for 3 h with imatinib (1 μM) and/or Akt inhibitor IV (1 μM). Phosphorylation of RPS6 was determined by Western blot analysis. Note that RPS6 was dephosphorylated with Akt inhibitor IV in both cell lines. Click here for file

Additional file 4 **Phosphorylation levels of STAT5, ERK1/2 and RPS6 in TKI- resistant cell line SUP-B15**. Cell line SUP-B15 was treated for 3 h with/without imatinib (1 μM), Raf kinase inhibitor I (100 nM), OSU-03012 (20 μM), wortmannin (1 μM) and rapamycin (10 nM). Phosphorylation of STAT5, ERK1/2 and RPS6 was determined by Western blot analysis. Note that RPS6 was dephosphorylated with all three inhibitors of the PI3K/mTOR pathway. Click here for file
