# Supplementary figures and images for: BCR-ABL1-independent PI3Kinase activation causing imatinib-resistance
Source: J Hematol Oncol. 2011 Feb 7;4:6. doi: 10.1186/1756-8722-4-6 (PMC3041785; doi:10.1186/1756-8722-4-6)

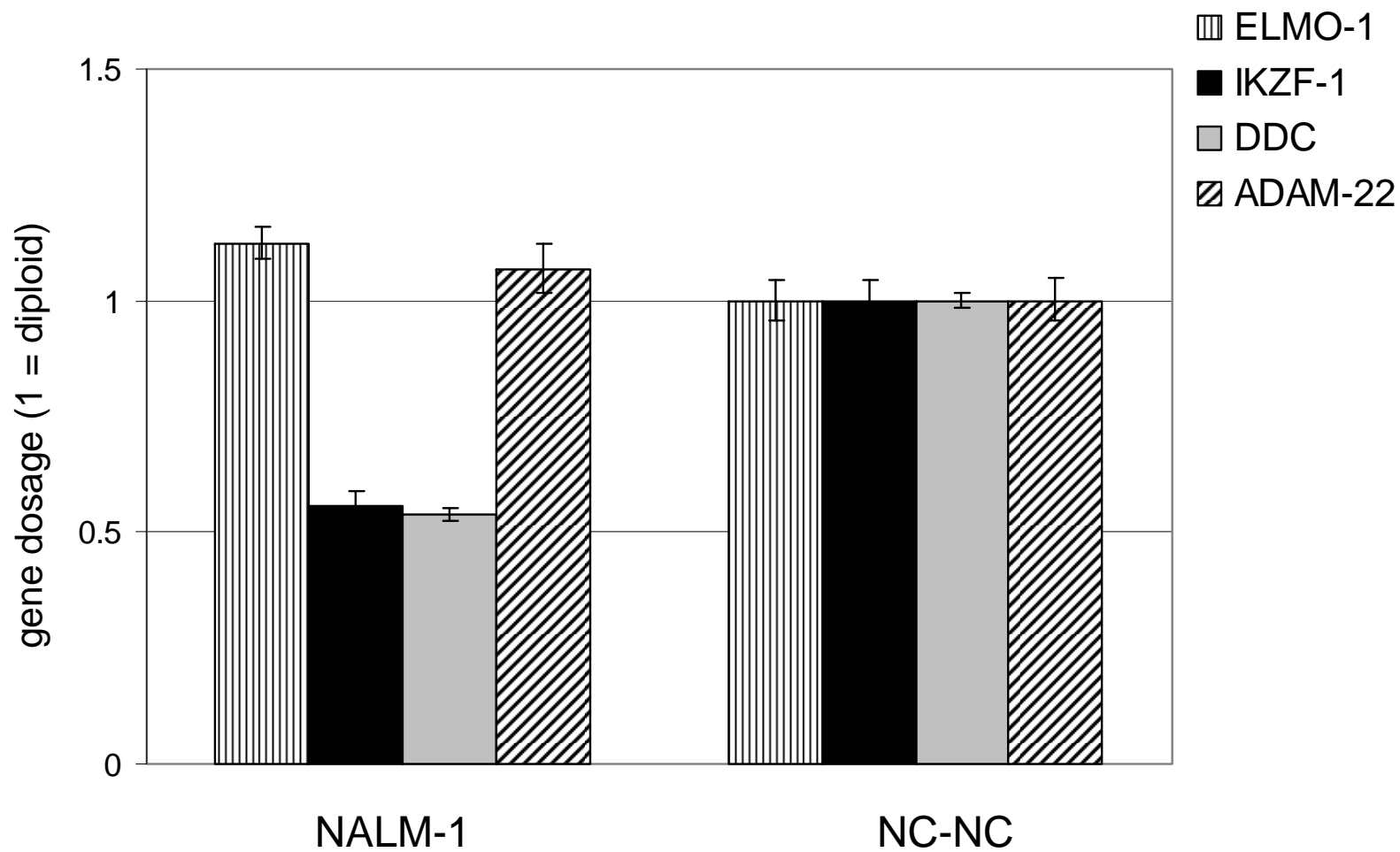

Supplement: Additional file 1 — Deletion of IKZF1 in cell line NALM-1. Quantitative genomic PCR confirmed loss of the genes IKZF1 and DDC, located between ELMO-1 and ADAM-22 at chromosome 7p12.2. The cytogenetically-verified diploid B-lymphoblastoid cell line NC-NC was used as reference, the repetitive element LINE1 was used as endogenous control. Cell lines SD-1, SUP-B15 and MHH-TALL1 did not show loss of IKZF1 according to quantitative PCR. [file 1756-8722-4-6-S1.PDF]

## SUP-B15 (resistant)

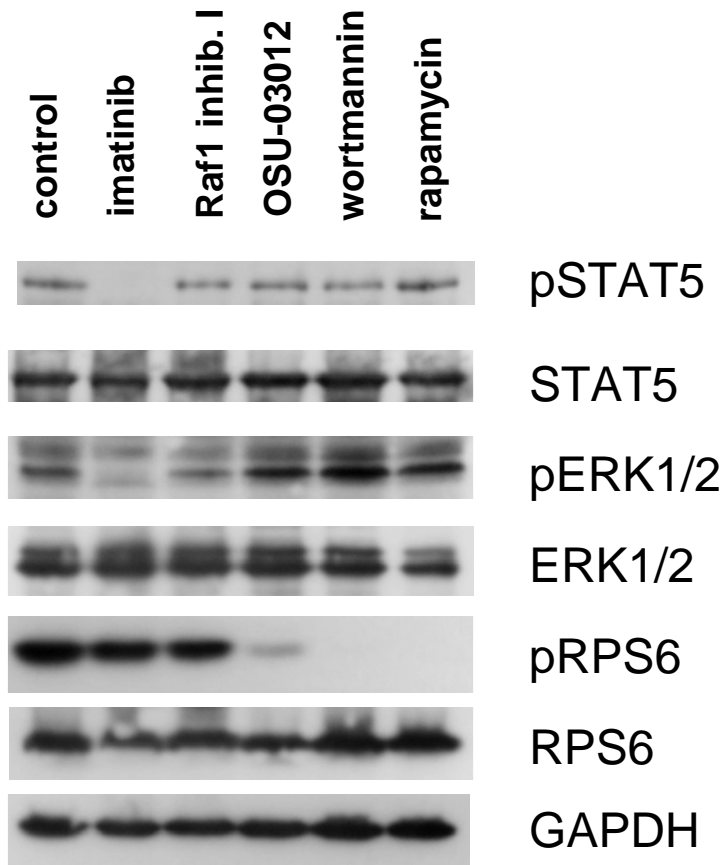

Supplement: Additional file 4 — Phosphorylation levels of STAT5, ERK1/2 and RPS6 in TKI- resistant cell line SUP-B15. Cell line SUP-B15 was treated for 3 h with/without imatinib (1 μM), Raf kinase inhibitor I (100 nM), OSU-03012 (20 μM), wortmannin (1 μM) and rapamycin (10 nM). Phosphorylation of STAT5, ERK1/2 and RPS6 was determined by Western blot analysis. Note that RPS6 was dephosphorylated with all three inhibitors of the PI3K/mTOR pathway. [file 1756-8722-4-6-S4.PDF]
