# Supplementary material for: BCR-ABL1-independent PI3Kinase activation causing imatinib-resistance
Source: J Hematol Oncol. 2011 Feb 7;4:6. doi: 10.1186/1756-8722-4-6 (PMC3041785; doi:10.1186/1756-8722-4-6)
Supplement: Additional file 2 — Phosphorylation levels of SRC, STAT5 and RPS6 in TKI-sensitive (JURL-MK2) and -resistant (SUP-B15) cell lines. Cell lines were treated for 3 h with the BCR-ABL1 and SRC kinase inhibitor dasatinib (20 nM) or with the SRC kinase inhibitor SU-6656 (2 μM) or control. Phosphorylation of the SRC kinases Fyn and Lyn, of STAT5 and RPS6 was determined by Western blot analysis. [file 1756-8722-4-6-S2.PDF]

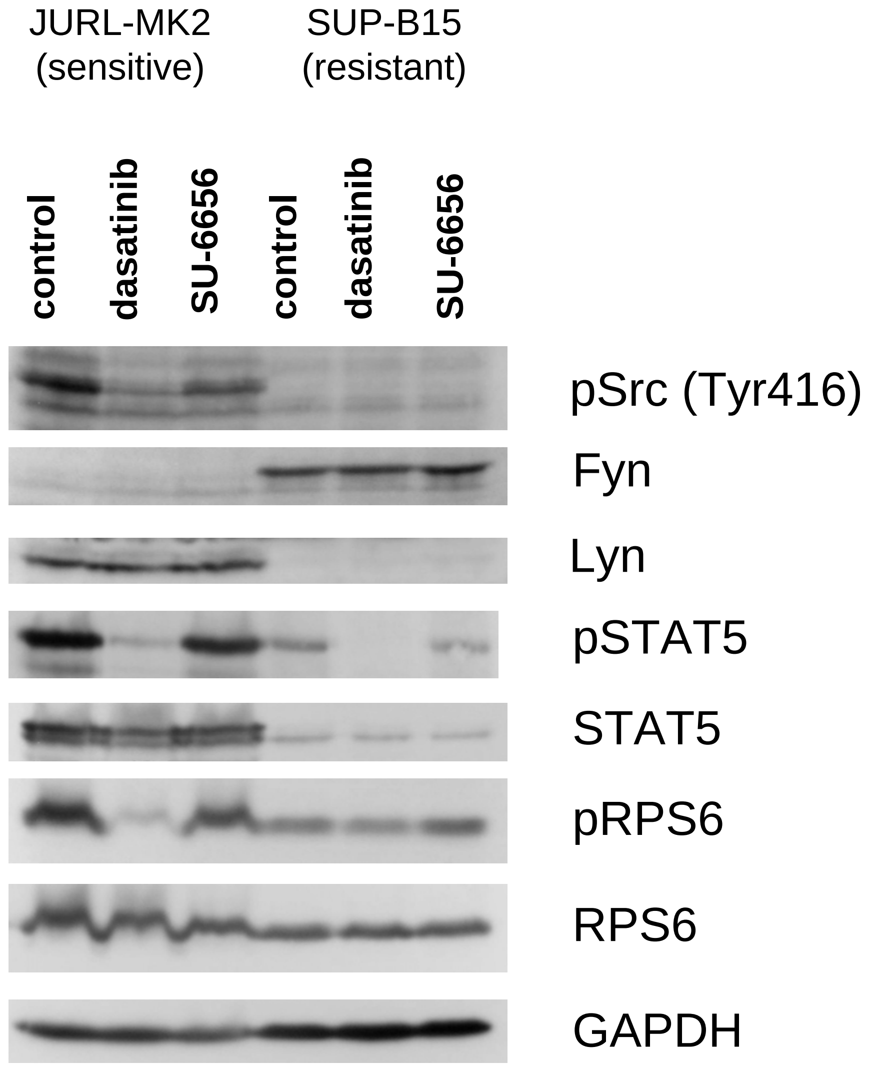
JURL-MK2
(sensitive)
SUP-B15
(resistant)
control
dasatinib
SU-6656
control
dasatinib
SU-6656
pSrc (Tyr416)
Fyn
Lyn
pSTAT5
STAT5
pRPS6
RPS6
GAPDH
