# Supplementary material for: BCR-ABL1-independent PI3Kinase activation causing imatinib-resistance
Source: J Hematol Oncol. 2011 Feb 7;4:6. doi: 10.1186/1756-8722-4-6 (PMC3041785; doi:10.1186/1756-8722-4-6)
Supplement: Additional file 3 — Phosphorylation levels of RPS6 in TKI-sensitive and -resistant cell lines. Cell lines were treated for 3 h with imatinib (1 μM) and/or Akt inhibitor IV (1 μM). Phosphorylation of RPS6 was determined by Western blot analysis. Note that RPS6 was dephosphorylated with Akt inhibitor IV in both cell lines. [file 1756-8722-4-6-S3.PDF]

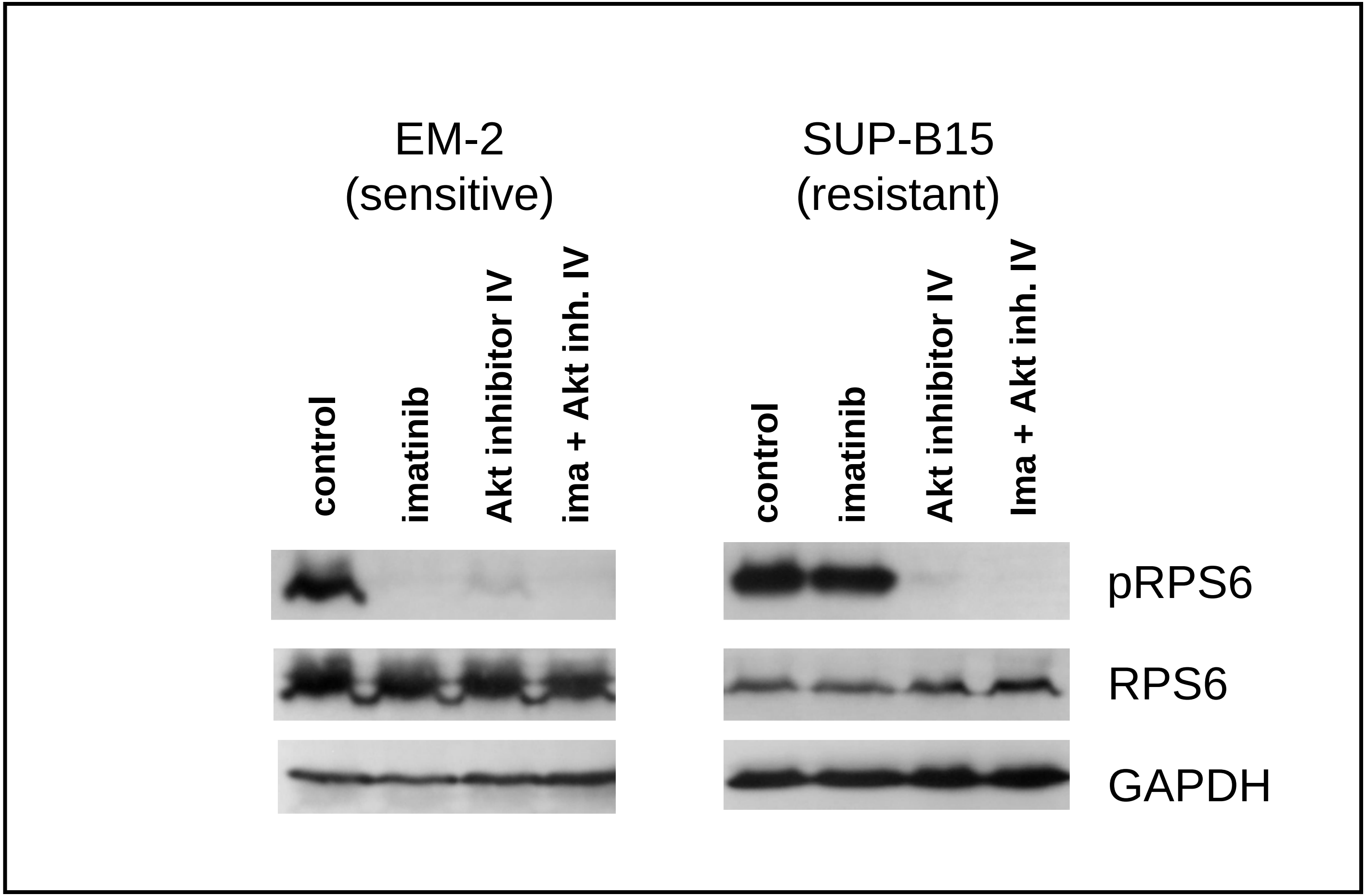

EM-2
(sensitive)
SUP-B15
(resistant)
control
imatinib
Akt inhibitor IV
ima + Akt inh. IV
control
imatinib
Akt inhibitor IV
Ima + Akt inh. IV
pRPS6
RPS6
GAPDH
